# Responses of Plasma Catecholamine, Serotonin, and the Platelet Serotonin Transporter to Cigarette Smoking

**DOI:** 10.3389/fnins.2019.00032

**Published:** 2019-03-04

**Authors:** Curtis Lee Lowery, Donna Woulfe, Fusun Kilic

**Affiliations:** ^1^Departments of Biochemistry and Molecular Biology, College of Medicine, University of Arkansas for Medical Sciences, Little Rock, AR, United States; ^2^Department of Biological Sciences, University of Delaware, Newark, DE, United States

**Keywords:** cigarette smoking, serotonin, catecholamine, platelet aggregation, serotonin transporter

## Abstract

Cigarette smoking is one of the major causes of coronary heart disease with a thirty percent mortality rate in the United States. Cigarette smoking acting on the central nervous system (CNS) to stimulate the sympathetic nervous system (SNS) through, which facilitates the secretion of serotonin (5-HT) and catecholamines to supraphysiological levels in blood. The enhanced levels of 5-HT and catecholamines in smokers’ blood are associated with increases in G protein-coupled receptor signaling and serotonylation of small GTPases, which in turn lead to remodeling of cytoskeletal elements to enhance granule secretion and promote unique expression of sialylated *N*-glycan structures on smokers’ platelets. These mechanisms enhance aggregation and adhesion of smokers’ platelets relative to those of non-smokers. This review focuses on the known mechanisms by which 5-HT and SERT, in coordinated signaling with catecholamines, impacts cigarette smokers’ platelet biology.

## Introduction

Serotonin (5-HT) is secreted from the enterochromaffin cells of the intestine to the blood and taken into the platelets by a specific transporter, SERT (SLC6A4) via a saturable reuptake mechanism (Rudnick, 1977; Lesch et al., 1993; McNicol and Israels, 1999; McNicol and Israels, 2003). Platelets do not synthesize 5-HT, yet they are the major biological storage pool of circulating 5-HT. Platelet surface located SERT tightly regulates the free 5-HT level in the blood. In platelets, vesicular monoamine transporter (VMAT) on the dense granules takes free 5-HT from the platelet cytoplasm into the dense granule which is sequestered there to millimolar concentrations, while free concentrations in the blood are in the low nanomolar range (McNicol and Israels, 2003; Brenner et al., 2007).

5-HT is a multifunctional signaling molecule, growth factor, and endocrine hormone or paracrine messenger (Maroteaux and Kilic, 2018) while it plays key roles in a variety of psychiatric diseases as a neurotransmitter, it also has extra-cerebral roles as a potent vasoconstrictor as well as a weak agonist of platelet activation, both roles serve to modulate cardiovascular disease (Rapport et al., 1984; Sjoerdsma and Palfreyman, 1990).

In various pathological conditions including hypertension and thrombosis, plasma 5-HT level is elevated (Biondi et al., 1986; Kerr et al., 1999; Vikens et al., 1999; Brenner et al., 2007; Davis et al., 2013; Fraer and Kilic, 2015; Lowery et al., 2017). The cause of 5-HT elevation, its effect on SERT, and the contribution to platelet physiology were studied in preclinical models, rat (Homberg et al., 2006) and mouse (Bengel et al., 1998) with SERT knock-out (KO) gene. These studies demonstrated that platelets were almost completely depleted of 5-HT in SERT-KO rodent models confirming that 5-HT in dense granules are also filled by platelet SERT through an uptake mechanism. Although the source of the initial elevation in plasma 5-HT levels remains controversial it has been proposed that it may originate from an increased rate of 5-HT synthesis or secretion from the enterochromaffin cells of the intestine due to the initial stage of a disease (Gershon and Tack, 2007). Studies with the blood samples collected from hypertensive subjects showed that there is a biphasic relationship with the plasma concentration of 5-HT and the density of SERT on platelet surface (Brenner et al., 2007). The level of SERT proteins on the surface of platelet and the 5-HT uptake rates of platelets initially rise as plasma 5-HT levels are increased, but then fall below normal as the plasma 5-HT level continues to rise (Brenner et al., 2007). These findings indicate a biphasic relationship between plasma 5-HT-level and the platelet SERT density on the plasma membrane, more specifically, down-regulation of platelet SERT in the presence of high level of 5-HT in plasma. We hypothesized that the elevated plasma 5-HT levels downregulate the 5-HT uptake of platelets via decreasing the density of SERT molecules on platelet surface. This hypothesis switches the cellular roles of 5-HT and SERT and proposes that 5-HT controls its own concentration in plasma by modulating the uptake properties of platelet SERT rather than SERT controls the plasma 5-HT levels. Further studies correlated the impact of 5-HT signaling on the membrane trafficking of SERT (Ahmed et al., 2008, 2009; Mercado and Kilic, 2010; Mercado et al., 2011).

In general, translocation of proteins from/to the plasma membrane is mediated by other proteins that facilitate their movement between the surface membrane and intracellular compartments. 5-HT signaling acts on the membrane trafficking of SERT molecules in two independent pathways: (1) on the exocytosis of SERT via acting on small GTPases (Ahmed et al., 2008; Li et al., 2016; Lowery et al., 2017; Mercado and Kilic, 2010; Mercado et al., 2011; Ziu et al., 2012); and (2) on the rate of internalization of SERT via acting on cytoskeletal proteins such as myosin IIa and vimentin (Ozaslan et al., 2003; Ahmed et al., 2009; Mercado and Kilic, 2010). The translocation of SERT from/to platelet plasma membrane is controlled by the 5-HT signaling-dependent pathways.

Studies in the platelets of mice lacking the gene for tryptophan hydroxylase (TPH1), the rate-limiting enzyme in the synthesis of 5-HT in peripheral cells, demonstrated that intracellular 5-HT acts on the exocytosis of the dense and α-granules during platelet activation (Walther et al., 2003; Ziu et al., 2012, 2014; Lowery et al., 2017). These studies in isolated platelets indicated that 5-HT-stimulation accelerated the exocytosis of granules, which secrete their contents, 5-HT, ADP, and procoagulant molecules, such as fibrinolytic regulators, growth factors, chemokines, immunologic modulators, P-selectin, von Willebrand factor, thrombospondin, fibrinogen, and fibronectin (Shirakawa et al., 2000; Walther et al., 2003; Ziu et al., 2012; Lowery et al., 2017). These findings specifically showed that 5-HT signaling manipulates the exocytosis mechanism of dense and α-granules in platelets. As explained in the following sections, the 5-HT signaling pathway controls the movement of SERT from/to plasma membrane of platelets (Ozaslan et al., 2003; Ahmed et al., 2008, 2009; Mercado and Kilic, 2010; Mercado et al., 2011; Ziu et al., 2012; Li et al., 2016; Lowery et al., 2017). This will elevate plasma 5-HT concentration to supraphysiological levels as seen during smoking cigarette (Lowery et al., 2017). However, smoking also elevates the blood plasma catecholamine levels (Siess et al., 1982; Grassi et al., 1994; Narkiewicz et al., 1998; Parati and Esler, 2012; Lowery et al., 2017). Together with 5-HT circulating catecholamines are associated with the increased risk of arterial thrombosis. Yet, in the absence of cardiovascular disease, elevated blood 5-HT level does not increase blood pressure, suggesting that the elevation in plasma 5-HT level could be a consequence rather than a cause of hypertension (Singh et al., 2013). Thus, the mechanisms by which elevated concentrations of 5-HT may lead to thrombosis in cigarette smokers is an active area of research that may yield improved development and application of anti-thrombotic therapy. In particular, the mechanisms by which 5-HT potentiates platelet aggregation may play a contributing role in the acutely increased risk of arterial thrombosis associated with cigarette smoking. The main goal of this review is to summarize a novel mechanism by which 5-HT and SERT in coordination with catecholamines impact cigarette smokers’ platelet biology.

### Cigarette Smoking and Clinical Relevance

Cigarette smoking has significant roles in the development of various cardiovascular diseases (CVD) through inhalation exposure of smoked tobacco and the secondary effects of tobacco products on circulating hormone levels. Even passive smoking (second hand smoke), with a smoke exposure about 1/10th that of active smoking, is associated with an approximate 30% increase of coronary artery disease (CAD), compared with an 80% increase in active smokers (Willett et al., 1987; He et al., 1999; Barnoya and Glantz, 2005). Despite increasing social and legal pressure to restrict tobacco use, an estimated 36.5 million adults in the United States still smoke tobacco cigarettes (Crow et al., 2018). Although awareness of these health risks has reduced the prevalence of smoking, a large portion of the population continues to use cigarettes and will suffer adverse cardiovascular events attributable to tobacco use.

While the exact toxic components of cigarette smoke and the mechanisms involved in cigarette-related long term cardiovascular dysfunction have not been fully elucidated, it has been repeatedly demonstrated that the acute effects (minutes to hours) of cigarette smoke are linked to plasma concentrations of nicotine (Powell, 1998; Mendelson et al., 2005; Tweed et al., 2012). Nicotine, a naturally occurring alkaloid found in the tobacco plant, appears to be the primary addictive and bioactive agent in cigarette smoke (Goldberg et al., 1991; Doolittle et al., 1995; Corrigall et al., 2001; Rabinoff et al., 2007). Inhalation results in rapid absorption of nicotine through the lungs into the blood stream. Nicotine crosses the blood-brain barrier and reaches the central nervous system (CNS) within 7 s of inhalation, where it stimulates nicotinic acetylcholine receptors (Doolittle et al., 1995; Mendelson et al., 2005; Sherva et al., 2008). Nicotinic CNS stimulation activates the sympathetic nervous system (SNS) to promote release of many chemical messengers including acetylcholine, adrenocorticotropin hormone (ACTH) norepinephrine (NE), epinephrine (E), arginine vasopressin, 5-HT, and dopamine (DA) (Koob and Le Moal, 2001; Kimes et al., 2003; Sherva et al., 2008; McKee et al., 2011) into blood plasma. Elevations in blood levels of these compounds are associated with the systemic cardiovascular effects of cigarette smoking (Ambrose and Barua, 2004; Mendelson et al., 2005).

Chronic cigarette smoking predisposes the individual to multiple atherosclerotic syndromes as well as peripheral atherosclerosis and aortic aneurysms (Winniford et al., 1987). Long term effects on lungs, blood vessels, and heart are well studied and have repeatedly shown that smoking cessation decreases the risk of all-cause mortality with an exponential decline approaching the risk of non-smokers at 5 years (Rosenberg et al., 1985; Fusegawa et al., 1999) Data also demonstrate an immediate reduction in thrombotic events following smoking cessation indicating important acute effects altering blood and platelet function (FitzGerald et al., 1988; US Department of Health and Human Services, 1990; Samet, 1991).

### Biological Mechanisms of Cigarette Smoking-Associated Thrombosis Risk

Thrombosis is the dysregulated formation of a thrombus, comprising the combination of platelet aggregates, and blood clot within blood vessels, such that the flow of blood through the circulatory system is obstructed. Under normal physiological conditions, regulatory mechanisms such as circulating levels of prostacyclin and endothelial expression of ecto-ADPase prevent thrombus formation. Without injury or other insult endothelial cells of intact vessels prevent blood clotting by secreting a heparin-like molecule and thrombomodulin. Additionally, the vascular endothelial cells prevent platelet aggregation and vasospasm by secreting nitric oxide. After vascular damage, thrombus formation is initiated to prevent blood loss after injury to a blood vessel. While acute injury results in hemostasis, which is the localized vascular response to prevent blood loss, thrombosis is a pathological response, typically initiated in the presence of atherosclerotic lesion. While hemostasis and thrombosis share similar initiating mechanisms, thrombosis proceeds to complete vessel occlusion, while physiological hemostasis stems blood loss, but allows maintenance of blood flow.

Hemostasis can be conceived as occurring via a three-step process (Levy et al., 2010). The first step is vascular spasm (vasoconstriction), caused by contraction of vascular smooth muscle cells. This local constriction of blood vessels results in decreased blood flow through the area and limits blood loss. At the area of damage, blood is also exposed to collagen in the subendothelial matrix. Collagen promotes adherence and activation of platelets localized at the site of injury. Once activated, platelets secrete dense and alpha granules, releasing platelet-activating factors, such as ADP and P-selectin, and causing the localized production of thromboxane A2 and thrombin on platelet and associated membrane surfaces. These factors activate other nearby platelets causing them to release their contents leading to a cascade effect (O’Connell, 2013). The activated platelets alter their shape through cytoskeletal remodeling, enhancing their adhesion to endothelial and other platelet surfaces and aggregate to form a platelet plug. Cytoskeletal remodeling is achieved predominantly through the modification of small GTP-binding proteins, resulting in downstream shape changes to a more spiny form with projecting filopodia. The increased surface area and alteration of surface proteins causes platelets to adhere to one another and to the exposed collagen in the damaged vessel wall. Signaling interactions then promote the activation of integrin alphaIIb-beta3 and subsequent binding of circulating fibrinogen, allowing formation of a stable platelet plug (Farndale et al., 2004). Coincident with these processes, coagulation is initiated on local membrane surfaces, allowing consequent formation of a clot. There is evidence that both platelet aggregation and clotting are excessively activated after acute exposure to cigarette smoke. Therefore, it is important to elucidate the mechanisms by which cigarette smoking induces platelets to become increasingly activated and increases thrombotic risk.

Cigarette smoking enhances the formation of thrombosis by which studies demonstrate that stopping smoking decreases the risk of the reoccurring of myocardial infarction (US Department of Health and Human Services, 1990). Also, epidemiologic evidence indicates that the acute effects of cigarette smoking produce CNS-mediated activation of the SNS (Siess et al., 1982; Peterson et al., 1983; Porchet et al., 1987; Narkiewicz et al., 1998; Parati and Esler, 2012), which stimulates secretion of pro-thrombotic molecules, such as catecholamines (E, NE, and DA) and 5-HT into the blood at supraphysiological levels (Koob and Le Moal, 2001; Kimes et al., 2003; Sherva et al., 2008; McKee et al., 2011). Recent studies from our laboratories demonstrate that smoking results in several-fold increases in plasma levels of 5-HT and catecholamines (Lowery et al., 2017). In platelets, 5-HT and catecholamine signaling are mediated, respectively, through 5-HT2A, β2-, and α2-adrenergic receptors. Activation of each of these receptors directly act on platelet aggregation (Woulfe, 2005; Millan et al., 2008; Smyth et al., 2009; Dowal and Flaumenhaft, 2010) via stimulating the exocytosis of the granules which are the storages of the several procoagulant molecules as well as 5-HT and catecholamine (McNicol and Israels, 1999, 2003).

Once bound to platelet surfaces, 5-HT activates a 5-HT-specific G protein-coupled receptor, 5-HT2A. Although the Gq-dependent signals leading to platelet activation are well-accepted, less appreciated is the coordinate interaction of these 5-HT and catecholamine-initiated signals with 5-HT uptake into the platelet cytoplasm. In this process, SERT, on the platelet plasma membrane, plays a major role in regulating extracellular vs intracellular 5-HT concentration. When 5-HT is removed from blood plasma into the platelet, it is stored in dense granules through VMAT (Brunk et al., 2006). Upon the saturation of dense granules with 5-HT, transport via VMAT shuts down and 5-HT appears in the cytoplasm in free, unbound form. The elevated plasma 5-HT level also activates platelet surface-localized 5-HT receptors, which initiates signaling through Gq, activates phospholipase C (PLC) results in the hydrolysis of phosphatidylinositol 4,5-biphosphate (PIP2) to inositol-1,4,5-triphosphate (IP3) (Jin and Kunapuli, 1998; McNicol and Israels, 1999; Yang et al., 2002; Quinton et al., 2004; Woulfe, 2005; Millan et al., 2008; Smyth et al., 2009; Dowal and Flaumenhaft, 2010; Bynagari-Settipalli et al., 2012). Formation of IP3 activates the serine/threonine protein kinase C (PKC) family and facilitates the secretion of Ca^2+^ from intracellular compartments to cytoplasm. Catecholamine (NE/E) activates α2-adrenoceptor which initiates signaling through Gi, inhibits adenylyl cyclase (AC) while activating phosphoinositide 3-kinases (PI3K), leading to the aggregation of platelets in a biphasic manner (Dowal and Flaumenhaft, 2010). Additionally, NE signaling also acts on Src family kinases (SFK) (McNicol and Israels, 2003; Walther et al., 2003; Senis et al., 2014) to allow PLC-dependent hydrolysis of PIP_2_ to IP3 and the secretion of Ca^2+^ from the granules (Walther et al., 2003; Ziu et al., 2012). Particularly, related to the role of 5-HT signaling in platelet pathology, elevation of the cytoplasmic free Ca^2+^ concentration activates transglutaminase (TGase), which transamidates unbound/free cytoplasmic 5-HT to small GTPases; a reaction known as serotonylation (Shirakawa et al., 2000; Walther et al., 2003; Ziu et al., 2012; Senis et al., 2014).

**FIGURE 1:**
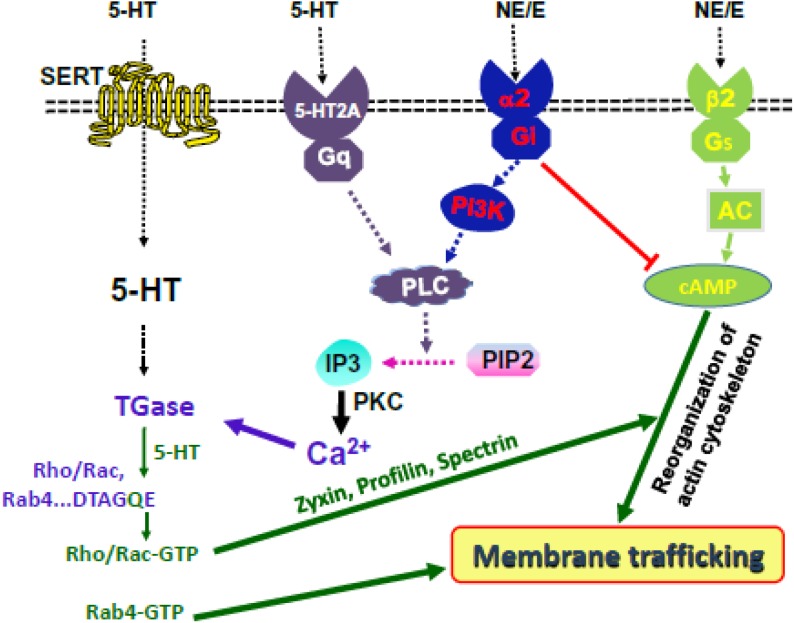
Hypothesis at a glance. 5-HT and NE signaling activate IP3 and elevate the level of cytoplasmic Ca^2+^. TGase serotonylates small GTPases. 5-HT and NE control platelet membrane-trafficking dynamics by enabling Rab4-GTP formation and Rho/Rac-GTP-mediated cytoskeletal rearrangement, downstream of cAMP/PKA.

The action of E/NE signaling on platelet via β2-receptor is a controversial topic. Yet, E/NE activates AC through the Gs, upregulates the intracellular concentration of the second messenger cAMP, a powerful inhibitor of platelet aggregation. The cellular act of cAMP reorganizes the actin/myosin cytoskeletal network in a Rho-GTP dependent manner via stimulating Protein kinase A (PKA) (Woulfe, 2005; Millan et al., 2008; Smyth et al., 2009). Thus, transamidation of Rho- and Rab-GTPases with 5-HT are important for platelet cytoskeletal reorganization and secretory behavior, respectively. We hypothesize that following cigarette smoking, IP_3_ is activated by 5-HT2A and α2-receptor signals to alter the cytoskeletal network in platelets and counteract the tonic inhibitory effect of β2-receptor signaling elevated cAMP level in platelets (Figure 1).

In the last decade, we reported several studies related with the impact of SNS-activated secretion of 5-HT into the blood (Brenner et al., 2007; Mercado and Kilic, 2010; Fraer and Kilic, 2015; Lowery et al., 2017), on platelet aggregation via activation of the 5-HT receptor (5-HT2A) (Ziu et al., 2014), on the secretion of granules as a result of serotonylation of small GTPases (Ahmed et al., 2008; Mercado et al., 2011; Ziu et al., 2012; Li et al., 2016), alteration of the cytoskeletal network (Ahmed et al., 2009; Li et al., 2016), and alteration of the structure and composition of glycans on the surface of mouse platelets (Mercado et al., 2013). Based on these studies, we proposed that 5-HT-mediated signaling and serotonylation of small GTPases accelerates the secretion rates of granule components such as ADP and P-selectin, remodeling of cytoskeletal network also permits altered localization of enzymes regulating glycosylation. The structures of glycans, specifically the terminal positions, act as ligands to the receptors. The best examples are P- and E-selectins which regulate various cell–cell adhesion in occurrence of vascular pathophysiology (Kelm and Schauer, 1997). Together, these studies show that the platelet surface glycans’ structures and distributions were altered by 5-HT treatment.

Our recently reported studies demonstrate that cigarette smoking induces specific changes to surface glycans and that these changes are associated with a potentiating effect on platelet aggregation (Lowery et al., 2017). Proteomic and glycomic analyses by Dr. Richard Jones at MS Bioworks and Dr. Parastoo Azadi at CCRC identified differences in both the number and specification of core proteins and glycans eluted from platelet plasma membranes isolated from nonsmokers versus smokers’ blood. Cigarette smoking acutely changes the glycan structure from high mannose to sialylated *N*-glycan structures expressed on platelet surfaces. Of particular functional relevance, removing the *N*-glycans from the surfaces of smokers’ platelets counteracted the smoking-mediated enhancements in platelet aggregation (Lowery et al., 2017), indicating that remodeling the platelet surface with *N*-glycan may establish a more adhesive environment and provide a mechanism by which smoking contributes to platelet activation and thrombosis. However, it remains to be investigated whether the high mannose structures present exclusively on resting (nonsmokers’) platelets protect against platelet aggregation. Specifically, proteins involved in the GTPase-activating, and associated with the actin cytoskeletal network were differentially and significantly altered on the surface of the smokers’ platelet compared to those of non-smokers.

These findings suggest that plasma 5-HT and catecholamine levels influence the alteration of glycans on platelet surfaces; however, the mechanisms by which 5-HT/catecholamine specifically induce surface glycan alteration is still not known. The abundance of oligomannose glycans on platelets suggests an attenuated N-glycan maturation pathway at the megakaryocyte level; however, the alteration in glycans on smokers’ platelet surfaces occurs within 15 min. This glycan structural alteration is too rapid to be explained by traditional glycan modification in Golgi apparatus after protein synthesis and subsequent completion of the membrane trafficking process (Lowery et al., 2017). Therefore, we propose that the involvement of 5-HT/catecholamine signaling in alteration of surface glycans could be through the membrane trafficking of several existing glycoproteins to the plasma membrane. In support of this proposal, *in vitro* studies of 5-HT/catecholamine-exposed nonsmokers’ platelets show an elevation in plasma glycan level and percent platelet aggregation rates to the levels found in smokers’ plasma, indicating a rapid additive effect of 5-HT and adrenergic receptors’ signaling. Furthermore, pharmacologic blockade of 5-HT and adrenergic receptors reduces both plasma glycan and platelet aggregation levels to that of non-smokers’ plasma (Lowery et al., 2017).

### In Summary

We propose that following cigarette smoking-activated 5-HT as well as α2-receptors downstream elements, such as IP_3_ hydrolysis-associated cytoplasmic level of free Ca and the cytoskeletal network in a cooperative manner to counteract the inhibitory effect of cAMP in platelet activation. Therefore, we propose that smoking-associated high levels of 5-HT and catecholamine in blood plasma make platelets prone to aggregation, in part, by changing the cytoskeletal network to accelerate the movement of *N*-glycan to the platelet surface.

## Author Contributions

This review article is a short summary of the studies performed by CL in FK’s laboratories during his Ph.D. program. DW has involved in the project through her expertise in G-protein coupled receptors on platelet.

## Conflict of Interest Statement

The authors declare that the research was conducted in the absence of any commercial or financial relationships that could be construed as a potential conflict of interest.
